# Supplementary material for: Chromosome-level genome assembly of a regenerable maize inbred line A188
Source: Genome Biol. 2021 Jun 9;22:175. doi: 10.1186/s13059-021-02396-x (PMC8188678; doi:10.1186/s13059-021-02396-x)
Supplement: Supplementary file 12 — Additional file 12. Review history. [file 13059_2021_2396_MOESM12_ESM.docx]

Review History

**First round of review**

**Reviewer 1**

**Are you able to assess all statistics in the manuscript, including the appropriateness of statistical tests used?** Yes, and I have assessed the statistics in my report.

**Comments to author:**

The manuscript (GBIO-D-20-01859) by Lin et., al. presented a chromosome-level genome assembly of an inbreed maize A188. Comparative genomics analysis between A188 and B73 revealed a couple of genetic variations associated with important traits, such as seed color. The authors also identified the expression and methylation patterns in embryonic callus comparing to other tissues. Overall, this genome assembly is of high-quality and the genomics analysis will provide useful information to the community. Even though, I have some suggestions that may improve the manuscript.
The genome was assembled by incorporating a number of technologies, including ONT, Illumina and BNG. However, no sequencing information of BNG was introduced in the METHODS section. What is the data size of BNG sequencing and what are the statistics of BNG assembly? In addition, the authors should provide more details of the genetic maps they used for chromosome construction.
In addition to B73 reference genome, there are a couple of maize genomes published, for instance, Mo17 and SK. Is it possible to include these published maize genomes in the genomics comparison with A188 to identify more variants associated with functional genes?
Line234, "Among landraces, both the A188 and B73 haplotypes of all six inversions could be identified". Did the authors mean that all of the six inversions identified in the comparison between A188 and B73 can also be found in other landraces? Any figure to support this conclusion?
Line 242, what does PME stand for?
Line 272, "A188 seeds are white, whereas B73 seeds are yellow". Through QTL mapping and comparison between the two genomes, the authors concluded that ccd1 and y1 genes likely contributed to the color difference, which was also supported by qRT-PCR. I am wondering is it possible to test the content of carotenoids in the two maize lines. The metabolism will add more evidence to explain the color difference.
The most interesting part to this reviewer is the expression and methlyation patterns in callus. The authors reported dynamic changes in the two aspects. I would expect more details what kind of genes/pathways are involved in the development of callus in A188, making this inbred line a good model for regeneration.

**Reviewer 2**

**Are you able to assess all statistics in the manuscript, including the appropriateness of statistical tests used?** Yes, and I have assessed the statistics in my report.

**Comments to author:**

The maize inbred line A188 is attractive with the features of highly embryogenic callus induction and transformation. Here, the genome is sequenced, assembled and annotated by using long-read sequencing of PacBio and Nanopore, combined with optical DNA mapping, which provide another high-continuity maize assembly and an invaluably functional resource. The authors also did the comparative genomics between B73 and A188 in terms of syntenic sequences and structural variations. They further studied gene copy number variation, especially identified the mechanism of white seed phenotype because of high ccd1 expression and low expression of y1 in seeds. There is no doubt that the genome will facilitate the finding of maize genetic diversity and understand associated phenotypic variation.

Nowadays, there are already many maize genomes sequences with third generation long read technology, such as a nested association mapping population containing 25 genomes (https://nam-genomes.org) and other 23 maize genomes (https://maizegdb.org/genome#!). The strategy of genome sequencing and assemblies is becoming routine and not novel anymore, while how to decipher the unique biological feature is critical to those genomes. Here, it was claimed that A188 can serve as a model inbred line for the genetic dissection of many important agronomic traits, including transformability, heterosis, and plant-environment interactions, whereas these traits are not deeply analyzed after the availability of A188 genome sequence. The team took a lot of effort to discover the causal variation in kernel color, whereas mapping the candidate genes for active callus induction is more critical for this unique genome. Still, differential gene expression and hypermethylation in calli relative to seedlings are not enough to target any causal variant yet, although it might be correlated to stress and defense response. There are a couple of studies focusing on QTL mapping involved in controlling callus induction or regeneration. I suggest the team continue to clone the candidate loci with the previous defined major QTLs with the guidance of the high-quality assembled A188. I believe that it will greatly improve the novelty by understanding the association of structural variation with maize regeneration.

Major revision
1)     Please add the description of embryonic callus of A188. Why is the A188 phenotypically unique compared to other maize inbreds?
2)     Please experimentally map candidate genes controlling callus induction.
3)     Please experimentally validate how hypermethylation could improve callus induction.

**Authors Response**

**Point-by-point responses to the reviewers’ comments:**

Reviewer 1

#############################################################

Reviewer #1: The manuscript (GBIO-D-20-01859) by Lin et., al. presented a chromosome-level genome assembly of an inbreed maize A188. Comparative genomics analysis between A188 and B73 revealed a couple of genetic variations associated with important traits, such as seed color. The authors also identified the expression and methylation patterns in embryonic callus comparing to other tissues. Overall, this genome assembly is of high-quality and the genomics analysis will provide useful information to the community. Even though, I have some suggestions that may improve the manuscript.

[your comment] The genome was assembled by incorporating a number of technologies, including ONT, Illumina and BNG. However, no sequencing information of BNG was introduced in the METHODS section. What is the data size of BNG sequencing and what are the statistics of BNG assembly? In addition, the authors should provide more details of the genetic maps they used for chromosome construction.

[our response]: We thank the reviewer for pointing this out. A summary table (Additional file 1: Table S4) describing statistics of the BNG raw data, filtering data, and assembled BNG maps is now provided. We also added the data for the genetic map (BAgm.v01, Additional file 1: Table S5) that was used for the contig scaffolding and the data for the genetic map (BAgm.v02, Additional file 1: Table S13) that was used for QTL mapping.

[your comment] In addition to B73 reference genome, there are a couple of maize genomes published, for instance, Mo17 and SK. Is it possible to include these published maize genomes in the genomics comparison with A188 to identify more variants associated with functional genes?

[our response]: The comparison with other maize genomes (Mo17, SK, version 5 of B73, and other NAM founders) has been done using Syri. The outputs were deposited to the Github under the manuscript repository (https://github.com/liu3zhenlab/A188Ref1). The inversions discovered from all these comparisons have been summarized and presented in the manuscript (Additional file 1: Fig. S19-23).

[your comment] Line234, "Among landraces, both the A188 and B73 haplotypes of all six inversions could be identified". Did the authors mean that all of the six inversions identified in the comparison between A188 and B73 can also be found in other landraces? Any figure to support this conclusion?

[our response]: Genome assemblies of landrace lines are still not available. Haplotypes of A188 and B73 at each inversion region were inferred by using SNP genotyping data in the inversion region. Both haplotypes of each of the six inversions can be observed in landrace lines based on their SNP genotypes. To be clearer, the sentence has been revised to “Among landraces, both the A188 and B73 haplotypes of each of the six inversions could be identified based on SNP genotyping data, supporting that all these six inversions exist in landrace maize lines.”. The original figures “Additional file 1: Fig. S19 and S20” supported the statement. The new figure numbers are “Additional file 1: Fig. S24 and S25”.

[your comment] Line 242, what does PME stand for?

[our response]: PME stands for pectin methylesterase, which was first mentioned on Page 6: “The clusters of genes encoding pectin methylesterase (PME) were identified on an unanchored scaffold c04_002 (two clusters with 25 and 18 genes), on chromosome 4 (one cluster with nine genes), and on chromosome 5 (one cluster with five genes) (Fig. 4a).”

[your comment] Line 272, "A188 seeds are white, whereas B73 seeds are yellow". Through QTL mapping and comparison between the two genomes, the authors concluded that ccd1 and y1 genes likely contributed to the color difference, which was also supported by qRT-PCR. I am wondering is it possible to test the content of carotenoids in the two maize lines. The metabolism will add more evidence to explain the color difference.

[our response]: Thank the reviewer for the suggestion. We have measured carotenoid contents in seeds of A188, B73, and two DH lines with different allele combinations from either A188 or B73. The carotenoid levels of these lines are expected based on their y1 and ccd1 expression. This new result has been added to the manuscript.

[your comment] The most interesting part to this reviewer is the expression and methlyation patterns in callus. The authors reported dynamic changes in the two aspects. I would expect more details what kind of genes/pathways are involved in the development of callus in A188, making this inbred line a good model for regeneration.

[our response]:

In our manuscript, we identified callus featured genes that exhibited a higher expression compared to other tissues. A set of pathways, such as cell wall biosynthesis, defense activity, heme binding, transmembrane transport, and transcription regulation, are enriched in these featured genes. We have listed some genes in the previously submitted manuscript. In the revised manuscript, we further examined the callus featured genes on a previously identified QTL region. Some genes in the enriched pathways are around the QTL, such as a Baby boom homolog (Zm00056a020360), a Wuschel2 homolog (Zm00056a020673), an LBD homolog (Zm00056a020860). In the region defined by fine mapping (Salvo et al. 2018), three genes, including a lignin biosynthesis gene Zm00056a020775 that encodes a caffeoyl shikimate esterase (CSE) were identified (Additional file 1: Fig. S32). We found a MITE insertion on a B73 syntenic gene at the promoter region that was missing in the A188 CSE gene. Further validation is needed to verify gene function in the callus development of A188.

#############################################################

Reviewer 2

#############################################################

Reviewer #2: The maize inbred line A188 is attractive with the features of highly embryogenic callus induction and transformation. Here, the genome is sequenced, assembled and annotated by using long-read sequencing of PacBio and Nanopore, combined with optical DNA mapping, which provide another high-continuity maize assembly and an invaluably functional resource. The authors also did the comparative genomics between B73 and A188 in terms of syntenic sequences and structural variations. They further studied gene copy number variation, especially identified the mechanism of white seed phenotype because of high ccd1 expression and low expression of y1 in seeds. There is no doubt that the genome will facilitate the finding of maize genetic diversity and understand associated phenotypic variation.

[your comment] Nowadays, there are already many maize genomes sequences with third generation long read technology, such as a nested association mapping population containing 25 genomes and other 23 maize genomes. The strategy of genome sequencing and assemblies is becoming routine and not novel anymore, while how to decipher the unique biological feature is critical to those genomes. Here, it was claimed that A188 can serve as a model inbred line for the genetic dissection of many important agronomic traits, including transformability, heterosis, and plant-environment interactions, whereas these traits are not deeply analyzed after the availability of A188 genome sequence.

[our response]: We agree with the reviewer that most of the traits of A188 were not characterized at this point. We have, on the other hand, constructed a B73xA188 DH population, which will facilitate trait analysis. The identification of causal variants for traits will be accelerated with the availability of the A188 reference genome. This manuscript is intended to provide the resources for trait analysis, which, including callus development, take time for each trait and is beyond the intent of this manuscript. It is also true that many genomes of additional maize lines have been provided recently. Our A188 genome will be valuable to be combined with others for maize pangenome studies.

[your comment] The team took a lot of effort to discover the causal variation in kernel color, whereas mapping the candidate genes for active callus induction is more critical for this unique genome. Still, differential gene expression and hypermethylation in calli relative to seedlings are not enough to target any causal variant yet, although it might be correlated to stress and defense response. There are a couple of studies focusing on QTL mapping involved in controlling callus induction or regeneration. I suggest the team continue to clone the candidate loci with the previous defined major QTLs with the guidance of the high-quality assembled A188. I believe that it will greatly improve the novelty by understanding the association of structural variation with maize regeneration.

[our response]: The seed trait analysis did not take considerable effort but was fortuitous. Seed color was easy to assess. Callus analysis requires considerably more labor intense effort and, as noted above, is in progress.

Major revision

[your comment] 1). Please add the description of embryonic callus of A188. Why is the A188 phenotypically unique compared to other maize inbreds?

[our response]: Maize inbred line A188 has a mixed origin and does not belong to either of the two main heterotic breeding groups. Therefore, A188 possesses distinct origins from most inbred lines used in hybrid production in the US corn belt (Wang and Dooner 2006; Liu et al. 2003). In the Additional file 2: Table S1, we provided the detailed comparison of many traits with the reference B73.

Liu, Kejun, Major Goodman, Spencer Muse, J. Stephen Smith, Ed Buckler, and John Doebley. 2003. “Genetic Structure and Diversity among Maize Inbred Lines as Inferred from DNA Microsatellites.” Genetics 165 (4): 2117–28.

Wang, Qinghua, and Hugo K. Dooner. 2006. “Remarkable Variation in Maize Genome Structure Inferred from Haplotype Diversity at the Bz Locus.” Proceedings of the National Academy of Sciences of the United States of America 103 (47): 17644–49.

[your comment] 2). Please experimentally map candidate genes controlling callus induction.

[our response]: We agree that it is important as a longer range goal of this project to map candidate genes controlling callus development. Indeed, the co-authors Heidi Kaeppler and Shawn Kaeppler conducted the genetic mapping experiment and identified a 3 Mb region (Salvo et al. 2018). Sanzhen Liu group is also working on mapping the genes controlling callus development from other loci. We have used the newly assembled A188 genome sequence to accelerate our genetic study. In the revised manuscript, we examined the genes on the 3Mb region (Salvo et al. 2018), three genes are callus featured genes that were up-regulated in the callus as compared to other tissues. These three genes include a lignin biosynthesis gene Zm00056a020775 that encodes a caffeoyl shikimate esterase (CSE). We found a MITE (miniature inverted-repeat transposable element) insertion on the B73 syntenic gene at the promoter region but not on the A188 CSE gene (Additional file 1: Fig. S32). Functional validation is needed in the future study to verify the gene function in the callus development. Presentation of the genome assembly data provides a resource to others who are examining additional traits for which A188 provides a good source of variation.

[your comment] 3). Please experimentally validate how hypermethylation could improve callus induction.

[our response]: In this manuscript, we observed the elevated methylation in the callus as compared to the leaf. With the observation of up-regulation of defense-related genes in the callus, we proposed that the DNA methylation might be a protective mechanism to maintain genome stability of callus cells that are under a high-level stress. The reviewer has proposed an important experiment. However, our data did not indicate that hypermethylation can improve callus induction.

**Second round of review**

**Reviewer 1**

The manuscript has been substantially improved and addressed all of my concerns.

**Reviewer 2**

In this modified version, the authors added the information about callus featured genes that exhibited a higher expression compared to other tissues. They also added three potential candidates including a Baby boom homolog (Zm00056a020360), a Wuschel2 homolog (Zm00056a020673), an LBD homolog (Zm00056a020860) from the previously defined QTL region. In addition, the group found a MITE insertion on a B73 syntenic gene at the promoter region that was missing in the A188 CSE gene. The functional validation for CSE (Zm00056a020775) is underway. It is true that deciphering candidate genes for controlling callus development is a long goal. Here, the group has listed plans that they and their collaborators are working on mapping those genes either by the construction of a B73xA188 DH population or other strategies. I believe that the identification of causal variants for traits will be accelerated with the availability of the A188 reference genome. I am looking forward to the day when the somatic embryogenic culture and regeneration are easily accessible, it could eventually break the obstacle to the low efficiency of producing genetically modified maize.
